# The Incidence of Clubfoot in the Czech Republic: A Nationwide Epidemiological Study from 2000 to 2014

**DOI:** 10.3390/children10040714

**Published:** 2023-04-12

**Authors:** Klára Janatová, Tereza Nováková, František Lopot

**Affiliations:** 1 Sport Sciences-Biomedical Department, Faculty of Physical Education and Sport, Charles University, Jose Martiho 31, 16200 Prague, Czech Republic; flopot@seznam.cz; 2 Department of Physiotherapy, Faculty of Physical Education and Sport, Charles University, Jose Martiho 31, 16200 Prague, Czech Republic; tnovakova@ftvs.cuni.cz

**Keywords:** clubfoot, congenital talipes equinovarus, epidemiology, foot deformity, incidence

## Abstract

Clubfoot is one of the most common musculoskeletal birth deformities worldwide. The prevalence varies among individual countries and populations. There is a lack of nationwide incidence studies in Central Europe. We analyzed the incidence of clubfoot in the Czech Republic over 14 years. Patients born with clubfoot in the Czech Republic were identified using The National Registry of Congenital Anomalies. Demographic data were included. Data from 2000 to 2014 were collected and analyzed regarding gender and regional distribution. The study’s chosen time frame was grounded on the condition of the Czech industry. Following extensive transformations in 1989, the industry eliminated highly non-ecological operations with significant environmental impact and related health risks. The incidence of clubfoot during the study period was 1.9 (95% CI 1.8–2.0) per 1000 births; males comprised the majority (59%). The incidence significantly differed among individual regions of the Czech Republic (*p* < 0.001). The incidence in the Czech Republic was higher than in previous European studies. We found significant regional differences in incidence, which could indicate that there may be exogenous pathogenic factors. For this reason, we plan to follow up our work with an up-to-date study.

## 1. Introduction

Clubfoot (CF), also known as congenital talipes equinovarus (CTEV), is one of the most common musculoskeletal birth deformities, with a prevalence of between 0.6 and 6.8 per 1000 live births [1]. Around 80% of clubfeet occur as isolated birth defects and are considered idiopathic [2]. Foot deformity is either bilateral or unilateral, characterized by hindfoot varus, forefoot adductus, an augmented midfoot arch, and ankle equinus [3]. The prevalence of idiopathic CF is higher in males compared to females [4]. CF develops early in pregnancy and can be detected by ultrasound, usually around 20 weeks of gestation [5]; according to Keret [6], it can be diagnosed from 12 weeks of gestation.

Today, the gold standard treatment is the Ponseti method [7] because of its effectiveness and less invasive correction with excellent results. Dr. Ignacio Ponseti, an orthopedic surgeon at the University of Iowa in the United States, developed the method. The Ponseti method uses serial manipulation, casting, and the tendo Achilles tenotomy to correct CF and foot abduction brace (FAB) to prevent relapse. Further, strategies to treat the relapse, based on the child’s age, are included in the method. The treatment goal is to provide long-term correction with a completely functional foot without pain [2].

The biomechanics of the Ponseti method have been studied extensively, and research has shown that this approach is highly effective, with up to 95% success rates. Related studies have also shown that the Ponseti method leads to better long-term outcomes and fewer complications than traditional surgical approaches [8,9,10]. Recently, nearly all orthopedics have agreed that conservative treatment is the best choice to correct CF [11].

Treatment ideally starts within the first few weeks of life, and a musculoskeletal examination is performed before manipulation and casting. The gentle manipulation of the foot is followed by the application of a long leg cast, as described by Ponseti. The casts are changed every 5 to 7 days. The cavus deformity is corrected first by supinating the forefoot with direct pressure under the first metatarsal head. The cavus deformity and medial crease are corrected, in most cases, with a single cast. The hindfoot varus, equinus, and forefoot adduction are simultaneously corrected in the subsequent three casts by gently abducting the foot in supination while counterpressure is applied to the talar head. Mainly after the fourth cast, all deformities are corrected except for the remaining hindfoot equinus. The gradual stretching and repositioning of the muscles, tendons, and ligaments in the foot to correct the inward twisting and misalignment that characterizes clubfoot use the basic principles of tissue relaxation and creep. An Achilles tenotomy is performed to reduce the equinus if less than 10 degrees of ankle dorsiflexion is present. A final cast is applied and left intact for three weeks to heal the Achilles tendon. The 4-finger modified technique must be utilized for complex and atypical CF; they resist the classic Ponseti method. After the final cast is removed, the patient goes directly into a FAB, and CF stretching exercises are demonstrated for parents to prevent relapse. Different braces are available today, but all share the same principle of the shoes attached to a solid or dynamic bar. The brace is worn full time (23 h a day) for 3 months and then at a sleeping time until 4 years of age [2,12,13,14,15,16,17,18].

The etiology of CF remains unclear, but there is convincing evidence for a multifactorial etiologic model involving both genetic and environmental factors [1].

The discovery that mutations in the PITX1-TBX4-HOXC transcriptional pathway cause familial CF and vertical talus in a small number of families has provided new insights into the pathogenesis of CF [19]. While a genetic influence has been identified as a significant etiological factor of idiopathic CF, no specific genes responsible for the deformity have been discovered yet [20].

Environmental factors may play a role in the etiology of clubfoot too. Early amniocentesis (<13 weeks gestation) has been associated with a ten times higher rate of CF than midgestational amniocentesis or not having this procedure performed [21,22]. Environmental exposure to cigarette smoke in utero is another independent factor increasing the risk of a child having clubfoot [23,24,25,26]. The study by Honein et al. reported a relationship between smoking and clubfoot with an adjusted odds ratio of 1.34 for smoking only, 6.52 for a family history only, and 20.30 for combined exposure to smoking and family [25].

No nationwide epidemiological study has focused on CF in Central European countries in the last 30 years. A population-based study by Wang [27] assessed the prevalence of CF only in 4 regions in 3 different countries in Central Europe (Germany, Poland, and Switzerland). To update information about the incidence of CF in Central Europe, we performed a nationwide epidemiological study in the Czech Republic over 14 consecutive years.

## 2. Materials and Methods

### 2.1. Study Design and Participants

The Czech Republic, situated in Central Europe with a population of 10.6 million, has had the National Registry of Congenital Anomalies (NRCA) since 1965. It is a nationwide population registry maintained by the Institute of Health Information and Statistics of the Czech Republic (IHIS CR). NRCA records all congenital malformations detected in children up to 15 years of age, stillbirths, and fetuses during pregnancy. In 2000, the NRCA changed the data collection method; the information source is the National Registry of Reproduction Health (NRRH) and also the National Registry of Newborns (NRN). It has been proven by IHIS CR (Czech abbreviation ÚZIS ČR) that congenital malformations were underestimated until 1999 [28], which means that the use of the NRRH itself was insufficient.

All birth defects are coded according to the MKN-10 (English abbreviation ICD-10) coding system published by IHIS CR in 2018. It is the Czech version of the International Statistical Classification of Diseases and Related Health Problems (ICD-10) issued by the World Health Organization (WHO) in 2016. Clubfoot is assigned the MKN-10 code Q660 [29].

We reviewed the NRCA database for notifications of CF for 20 years—from 1994 to 2014. The reports contained information about congenital malformation diagnosis (MKN-10), the sex of the infant, and the year of birth. In addition, demographic data were collected. Healthcare establishments also provide: 1. Data on the fetus (completed gestation week at detection of congenital malformation, detection of congenital malformation (year, month, day), termination of pregnancy (year, month, day)); 2. Data on the newborn (birth weight (g), birth length (cm), and date of birth (year, month, day)); and 3. Additional data on the child (assisted reproduction, prenatal diagnostics, data on the mother (residence, marital status, occupation, etc.), congenital malformations in the family, infectious disease in the 1st trimester/in gestation week, the number of previous spontaneous/artificially induced abortions, and data on father) to the NRCA. The NRCA refused to share these data with us because of the General Data Protection Regulation. We received only the reports assigned with the diagnosis Q660 and the related information mentioned above (the sex of the infant, the year of birth, and demographic data).

We used data from 2000 to 2014 when the data were already collected from both registries (NRRH and NRN). Data from 1994 to 1999 were excluded; they are non-comparable with the data collected since 2000. It was not possible to exclude non-idiopathic cases (syndromic CF, positional CF). Thus, 2957 children were included in the study. However, 45 cases were excluded from the demographic part due to unknown data on the mother’s residence; therefore, finally, 2912 children remained. The laterality of deformity is not known.

### 2.2. Statistical Analysis

To compare the annual number of newborns with CF with the total number of live births in the Czech Republic from 2000 to 2014, we used official reports concerning native data from the Czech Statistical Office. The incidence was calculated as a ratio between the number of children with CF born between 2000 and 2014 and the number of live births during the same period. It is expressed as a rate per 1000 live births. The 95% confidence intervals (CIs) were calculated for the estimated incidence. CIs were assigned for the total number of cases and the subgroups—sex and region.

The country was divided into 14 regions, according to the Ministry of the Interior of the Czech Republic, to assess the geographical distribution of CF cases. This division is equivalent to the Nomenclature of Territorial Units for Statistics—Level 3 (NUTS 3), which Eurostat and other European Union bodies commonly use. The chi-square test was used to determine whether the regional incidences significantly differed.

Multivariate logistic regression with two explanatory variables (sex and region) was used to investigate the association between the risk factors (mother’s residence and sex of the infant) and CF. Adjusted odds ratios (ORs) and associated 95% CIs were calculated according to the following equation:

$$\mathrm{OddsRatio}\;\left(G1,R1\;\mathrm{vs}.\;G2,R2\right)=\frac{\mathrm{odds}\left(G1,R1\right)}{\mathrm{odds}\left(G2,R2\right)}$$

Gender (G1 = G2) with different regions (R1, R2) led to a comparison of regions within one gender, and the region (R1 = R2) with different genders (G1, G2) led to a comparison of the genders within the same region. Due to multiple tests being conducted at once, the threshold to which the *p*-value was compared was corrected according to the so-called Bonferroni correction when testing M tests at the 0.05 level compared to 0.05/M.

Data were entered into Microsoft Excel for Windows, and statistical analysis was performed using the free statistical software, R (version 3.6.1.). A *p*-value of < 0.05 was considered significant for all statistical tests.

## 3. Results

### 3.1. Incidence of Clubfoot in the Czech Republic

Between January 2000 and December 2014, 2957 cases of CF were identified from the NRCA in IHIS CR. Among the 1,577,365 newborns recorded in the Czech Republic during the study period, the incidence of CF was 1.9 (95% CI 1.8–2.0) per 1000 births (Table 1). The incidence each year of the 14 years studied ranged from 1.5/1000 to 2.3/1000. The lowest incidence (1.5/1000) was in 2014, and the highest was in 2000 (2.3/1000). The yearly incidence varied during the study period; it was not a simple decreasing trend.

### 3.2. Incidence of Clubfoot According to Sex

**Figure 1:**
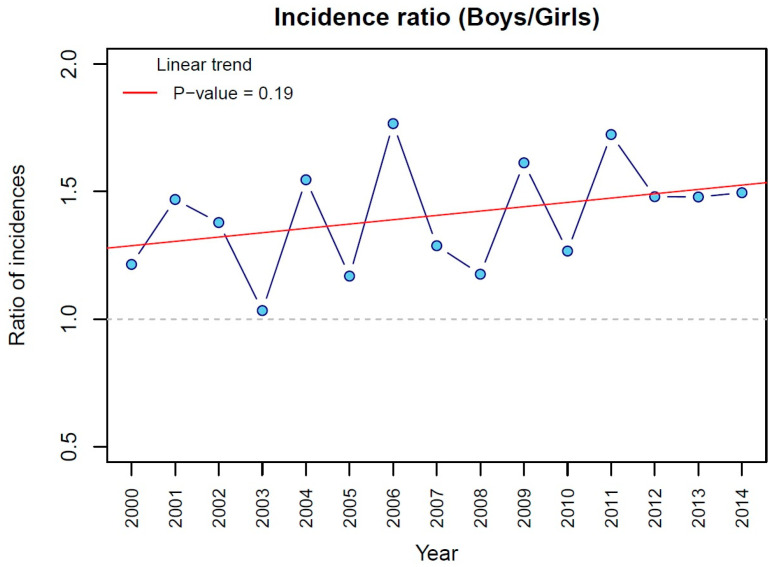
The incidence ratio between boys and girls in the years 2000–2014.

1755 males and 1202 females were diagnosed with CF. The majority (59%) of the cases were boys. The male: female ratio was 1.4 (95% CI 1.3–1.5), which was statistically proven to be different from 1 (*p* < 0.001), thus confirming the higher susceptibility of boys. The annually estimated incidence ratios (14 years study period) had a slightly increasing trend. However, this could not be statistically confirmed (*p* = 0.19), i.e., the incidence ratio between boys and girls remained constant over time (Figure 1). The incidence for boys was 2.2/1000 (95% CI 2.0–2.3) and 1.6/1000 (95% CI 1.4–1.7) for girls during the 14-year span (Table 2).

### 3.3. Incidence of Clubfoot According to Geographical Distribution

**Figure 2:**
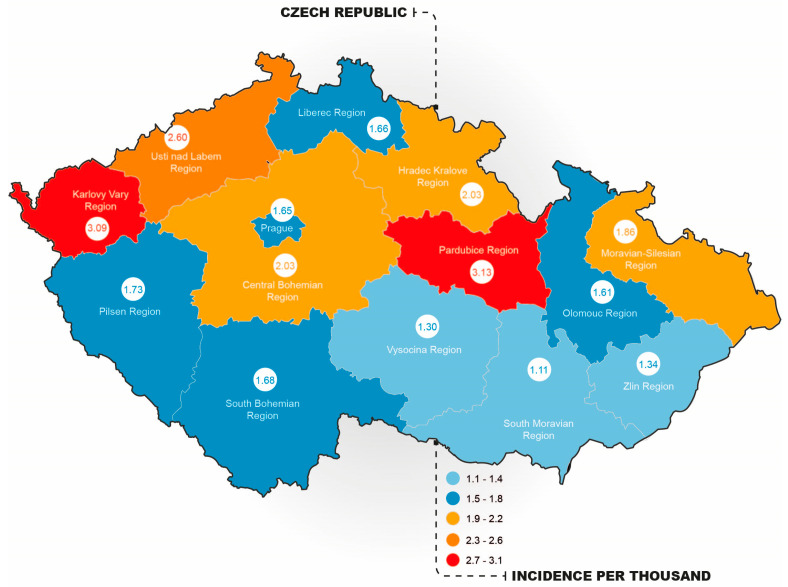
Incidence of clubfoot during 2000–2014 in the Czech Republic (14 regions—NUTS 3).

The use of the chi-square test demonstrated significant differences (*p* < 0.001) in regional incidences, as shown in Table 3 and Figure 2. The highest incidence of 3.1/1000 was found in two regions, the Pardubice Region (95% CI 2.5–3.8) and the Karlovy Vary Region (95% CI 2.4–3.8), while the lowest incidence of 1.1/1000 was observed in the South Moravian Region (95% CI 0.9–1.3).

Multivariate logistic regression revealed a statistically significant association between CF and maternal residence. The lowest incidence was, in general, in the South Moravian Region. Boys born in the Karlovy Vary Region showed the highest incidence at 2.6 times (95% CI 1.8–3.4) higher compared to the South Moravian Region. In comparison, the highest incidence in girls was in the Pardubice region, which was 3.1 times (95% CI 2.3–4.0) higher compared to the South Moravian Region.

When combining both sexes, the Pardubice region had the highest risk of CF compared to the South Moravian Region, with roughly 2.8 times (95% CI 2.3–3.4) higher incidence.

## 4. Discussion

This study aimed to investigate the incidence and geographical distribution of CF in the Czech Republic over a long period of time. There is an overall lack of epidemiological studies in neighboring countries. The study’s chosen time frame was grounded on the condition of the Czech industry. Following extensive transformations in 1989, the industry eliminated highly non-ecological operations with significant environmental impacts and related health risks.

We found a higher incidence of congenital CF during the 14 years of the study than has been found in previous studies conducted in Europe: 1.03/1000 live births in Sicily [30], 1.1/1000 live births in Norway [24], 1.13/1000 live births in 18 regions in Europe [27], 1.2/1000 live births in Denmark [31], and 1.4/1000 live births in Sweden [32].

Underreporting is often one of the issues of registry studies. Therefore, it naturally spawns the question of whether there is a problem with overreporting or if the Czech registry may be more accurate than the others. Most children in the Czech Republic are born in hospitals, and CF is easily diagnosed at birth by health professionals. The congenital malformation is reported by the physician who diagnosed it and specifically names it, not by a doctor who only suspects it. It is reported to the NRRH. Further, every neonatal department or children’s and female department is responsible for completing the mandatory “Newborn Report” to the NRN. In the case of a birth outside of a health care facility (birth at home, in a means of transportation, in public areas, etc.), the health care worker who assisted during labor or performed the first postpartum treatment of the woman and the newborn is obliged to report it too. Since the NRCA added the NRN to the data collection system in 2000, we think it should mitigate underreporting issues.

Overreporting of children with idiopathic CF may still occur, for example, in the fully reducible foot with no need for regular treatment (positional CF) or complex forms with additional malformations of neurological origin [33]. We could not focus on only idiopathic cases because we could not withdraw syndromic and posinitional CF. Further, other diagnoses may be reported as a CF deformity (metatarsus varus foot, calcaneovalgus foot, etc.). We considered it a limitation of our study that we could not examine hospital records in addition to registry reports, as in other studies [31,32]. Thus over-estimation of CF diagnosis may be present. Since 2009 there has been indirect pressure on healthcare system professionals in the Czech Republic from the patients’ organization, Achilleus, which serves kids and adults diagnosed with CF in the Czech Republic. The organization informs parents and the general public about this foot malformation and its treatment possibilities, which also increases awareness of CF among healthcare system professionals. So far, we cannot say whether this indirect pressure could improve CF recognition over other foot deformities.

The reported incidence varies across the world, and it is difficult to compare the results regarding the complicating factors, such as variations in diagnostic criteria and the registration methods used [34]. The world population is changing not only over centuries but every year. The population change is influenced by several parameters (population size, density, birth/death rates, etc.). Therefore, from our perspective, epidemiological studies from the 20th century are losing their value.

The fact that a study was published after the turn of the millennium does not mean that the majority of data were collected in that period. Wallander [32] collected data from 1995 to 1996; data from the registry in Denmark were collected from 1978 to 1993 [31]; the study period in Sicily was from 1991 to 2004 [30]; in the United Kingdom, hospital records were collected from 1992 to 2006 [35]. Carey [36] collected data from 1980 to 1994 in Western Australia, Moorthi [37] from 1996 to 1999 in Texas, Byron-Scott [38] from 1986 to 1996 in South Australia, etc. This could be one of the reasons why the incidence of CF was higher in our study: data collection was from a different period.

The mean incidence in the Czech Republic in 1994–1999 was 1.3/1000 (unpublished data), but it is the output of different methods of collecting data. We impute these findings as insufficient reporting to the NRCA. This incidence is in accordance with the findings of Wallander and Krogsgaard [31,32].

### 4.1. Incidence over Time

Some studies [31,32] declare increasing incidence over time, but we could not confirm these authors’ observations. The incidence in the Czech Republic fluctuated during the study period. The incidence increase in Denmark was seen mainly in isolated CF, not in syndromic ones [31]. On the contrary, a decreasing trend was found in the study, using data from 18 EUROCAT registries in Europe [27].

### 4.2. Boys vs. Girls

Our study supported previously reported findings [23,36,39] that male sex is a strong risk factor.

The incidence of isolated CF in Denmark was 2.2 times higher in boys than in girls; however, for syndromic CF, the ratio was only 1.4 [31], which is the same as our findings for all types of clubfeet. Krogsgaard mentions that this suggests that CF is a multi-genetic disease: partially related to sex chromosomes and partially to information on other chromosomes and exogenous factors. Our male/female ratio does not correspond with those of other authors, who have reported the ratio as 2–2.5 times higher in boys compared to girls [4,37]; however, both studies only compared idiopathic CF.

We found out that the risk of having CF is higher in boys than in girls in the Czech Republic. According to the information from the IHIS CR (unpublished data), the risk of having any congenital malformation is also significantly higher in boys than in girls. A large part of this difference is due to the frequency of congenital malformations of the genitals. These defects represented 19.5% of all congenital disabilities in boys and only 1.3% of all defects diagnosed in girls in 2011. Even if we did not count the birth defects of genital organs, the number of boys born with a congenital defect would be still higher than in girls [40]. We do not know any other reason why congenital malformations occur more often in the male sex.

### 4.3. Geographical Distribution

However, the regional differences in the incidence of CF may play a role. This has been confirmed in our study and also in studies from Wallander, Moorthi, and Parker [32,37,41]. They observed diversity in the incidence of CF based on the region of residence, and some of them also with respect to ethnicity. Significant regional differences were found in isolated CF in Europe using data from the EUROCAT registries [27]. Cases from 18 registries were included, and the incidence (CF cases without chromosomal anomaly) ranged from 0.44/1000 births (95% CI 0.37–0.52) in Tuscany, Italy to 1.68/1000 births (95% CI 1.57–1.80) in Wales, United Kingdom. In Parker’s study [41] across the USA, the highest incidence was in Colorado (1.73/1000) and the lowest was in West Virginia (0.95/1000).

We found statistically significant differences between several regions in the Czech Republic (Table 3, Figure 2). The highest incidence was in the Northwest (Karlovy Vary Region, Usti nad Labem Region) of the Czech Republic and the Pardubice Region. These findings are in concordance with the IHIS CR because these three regions have the highest incidence of congenital malformations in the Czech Republic annually (observed period 2000–2014). Further, the Liberec Region and the Hradec Kralove Region are on the higher end of the scale of congenital malformations within the Czech Republic. A study from Šípek presented data from 1994 to 2007 when the highest incidences of birth defects in the Czech Republic regions were in Karlovy Vary, Hradec Kralove, and Pardubice regions [42]. That makes the Northwest and Northeast parts (the Liberec, Hradec Kralove, and Pardubice regions) of the Czech Republic the areas with the highest incidence of congenital malformations of any kind.

**Figure 3:**
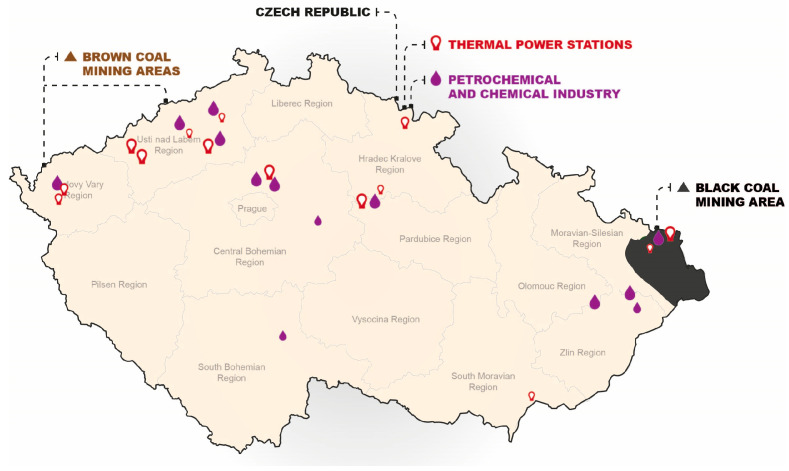
Regions of the Czech Republic (NUTS 3) and coal mining areas, thermal power stations, and petrochemical and chemical industries.

The Northwest part is well known because of the “Black Triangle“and its history of high levels of air pollution (Figure 3). The “Black Triangle“ is a border zone area between the Northwest Czech Republic, former East Germany, and Southwest Poland. There are inefficient heavy industry stations and power plants fuelled by poor-quality lignite (brown coal with high sulfur and ash content). The severe impacts on the population’s health in the valleys in this region have been well-documented [43].

Air pollution in this region improved after the “Velvet revolution “in 1989 [44] after establishing the Ministry of the Environment of the Czech Republic and introducing air pollution legislation convergent with European Union priorities [45]. However, the results of a higher incidence of CF may also be influenced by family history. It has been proven that family history plays an important role in the etiology of CF [46]. According to Honein et al. [25], environmental exposure to cigarette smoke in utero, in the presence of a positive family history of CF, increases the risk by 20 times. The study by Huang et al. examined the ethnic distribution of major birth defects and the relationship between air pollution and birth defects in Liuzhou City, China. The study found that congenital disabilities, including CF, were positively associated with air pollutants PM10 and CO in the second and third months of pregnancy. The study provides further evidence of the association between air pollution exposure during gestation and birth defects.

**Figure 4:**
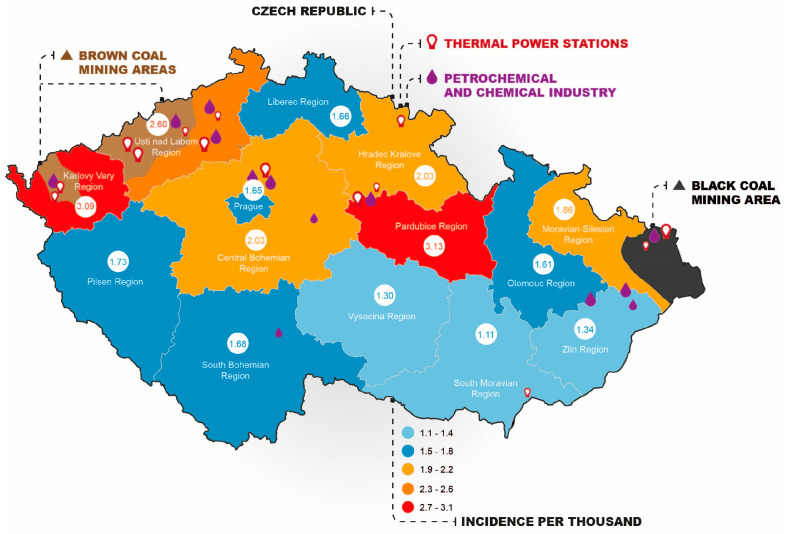
Incidence of clubfoot (during 2000–2014) in 14 regions of the Czech Republic (NUTS 3) and coal mining areas, thermal power stations, and the petrochemical and chemical industries.

Pardubice Region is known for its chemical and petrochemical industries and mechanical engineering (Figure 3 and Figure 4). Various other industrial activities, refineries, and petrochemical industries can affect air quality; they may release pollutants, such as VOCs, particulate matter, and greenhouse gases [47]. The leading refinery and petrochemical group is Unipetrol, located in Pardubice (Pardubice Region) and Litvinov (Usti nad Labem Region). We think that chemical factories and oil refineries could be one of the reasons for the high incidence of CF in this region. Exogenous factors can play a role in the etiology of CF [31], and Pardubice has been an industrial city for the last 100 years.

A significant increase in the incidence of CF has been connected with population density regardless of sex [31]. These incidence rates suggest that exogenic factors, such as environmental stress (pollution, traffic) and stress of urban living (alcohol, tobacco, and drug abuse) are more common in densely populated areas. We could not confirm or disprove this, but it could be further explored.

We did not examine the regions of the Czech Republic with regard to ethnicity, which has been performed in other studies [37,41]. The majority (approx. 95%) of the inhabitants of the Czech Republic are ethnically and linguistically Czechs, but there are other ethnic groups, including Germans, Gypsies, Poles, Vietnamese, and Hungarians. It is not known whether ethnicity could play a role in the difference in the incidence across the country.

### 4.4. Limitations of the Study

Our study has several limitations. It is a registry study, and only primary data (the sex of the infant, the year of birth, and demographic data) were provided to us. NRCA refused to share the other data with us, and we could not obtain any hospital records to find out this information. Unfortunately, we had no data about the pregnancy and the newborn presentation. Further, we did not have any information about the CF deformity, in terms of whether it was idiopathic (isolated deformity) or syndromic (other underlying diagnosis or syndromes), its laterality, or its classification (Pirani, Dimeglio).

There is uncertainty in the physician-reported CF diagnosis. We acknowledge that this may result in inaccurate reporting, which could lead to overreporting or underreporting.

## 5. Conclusions

Even though CF is one of the most common musculoskeletal birth defects, there is still much unknown regarding its etiology, and information about incidence is not often updated. Epidemiological studies published in the second half of the 20th century are losing their value. Our findings emphasize the importance of recent nationwide epidemiological studies and their usefulness in identifying the exact etiology of CF. It may help determine the prognosis and the selection of appropriate treatment methods, maybe even prevent this common congenital malformation. We have conducted the first nationwide epidemiological study in Central Europe and one of the recent incidence studies that collected data after the year 2000.

Despite that, establishing a uniform registry over Europe, including complete medical information, would be the logical step to improve nationwide data collection, processing, and evaluation.

## Figures and Tables

**Table 1:** The number of children with clubfoot in relation to the number of native births.

Year	No. of Children with Clubfoot	No. of Children Born Alive	Incidence per Thousand
2000	205	90,910	2.3
2001	194	90,715	2.1
2002	150	92,786	1.6
2003	182	93,685	1.9
2004	161	97,664	1.6
2005	183	102,211	1.8
2006	173	105,831	1.6
2007	213	114,632	1.9
2008	235	119,570	2.0
2009	217	118,348	1.8
2010	241	117,153	2.1
2011	217	108,673	2.0
2012	232	108,576	2.1
2013	189	106,751	1.8
2014	165	109,860	1.5
Total	2957	1,577,365	1.9 (95% CI 1.8–2.0)
Boys	1755	809,676	2.2 (95% CI 2.0–2.3)
Girls	1202	767,689	1.6 (95% CI 1.4–1.7)

**Table 2:** Incidence of clubfoot—boys and girls.

Year	Boys Incidence per Thousand	Girls Incidence per Thousand
2000	2.5	2.0
2001	2.5	1.7
2002	1.9	1.4
2003	2.0	1.9
2004	2.0	1.3
2005	1.9	1.6
2006	2.1	1.2
2007	2.1	1.6
2008	2.1	1.8
2009	2.3	1.4
2010	2.3	1.8
2011	2.5	1.5
2012	2.5	1.7
2013	2.1	1.4
2014	1.8	1.2
Total	2.2 (95% CI 2.0–2.3)	1.6 (95% CI 1.4–1.7)

**Table 3:** Incidence of clubfoot (95% CI) during 2000–2014 in 14 regions of the Czech Republic.

	Region	Native Births	Clubfoot Children	Incidence in Total	Incidence Boys	Incidence Girls
1.	Prague	187,934	310	1.6 (1.4–1.9)	2.1 (1.8–2.5)	1.1 (0.9–1.4)
2.	Central Bohemian	194,935	396	2.0 (1.9–2.2)	2.4 (2.1–2.7)	1.6 (1.4–1.9)
3.	South Bohemian	95,003	160	1.7 (1.5–1.9)	2.1 (1.8–2.3)	1.3 (1.0–1.6)
4.	Plzeň	83,432	144	1.7 (1.4–2.1)	1.9 (1.4–2.4)	1.5 (1.0–2.0)
5.	Karlovy Vary	45,936	142	3.1 (2.4–3.8)	3.2 (2.2–4.1)	3.0 (2.0–4.0)
6.	Ústí nad Labem	130,558	340	2.6 (2.2–3.0)	3.0 (2.6–3.5)	2.2 (1.7–2.6)
7.	Liberec	68,136	113	1.7 (1.3–2.0)	1.9 (1.3–2.4)	1.4 (1.0–1.8)
8.	Hradec Králové	82,579	168	2.0 (1.6–2.4)	2.5 (2.0–2.9)	1.6 (1.0–2.2)
9.	Pardubice	77,365	242	3.1 (2.5–3.8)	3.1 (2.6–3.7)	3.1 (2.2–4.0)
10.	Vysočina	76,164	99	1.3 (1.1–1.5)	1.6 (1.3–1.9)	1.0 (0.6–1.4)
11.	South Moravian	174,288	194	1.1 (0.9–1.3)	1.2 (1.0–1.5)	1.0 (0.8–1.2)
12.	Olomouc	94,688	152	1.6 (1.3–1.9)	1.7 (1.3–2.2)	1.5 (1.2–1.8)
13.	Zlín	84,051	113	1.3 (1.1–1.6)	1.6 (1.3–2.0)	1.0 (0.8–1.3)
14.	Moravian-Silesian	182,296	339	1.9 (1.6–2.1)	2.1 (1.8–2.4)	1.6 (1.2–1.9)

## Data Availability

The data presented in this study are available upon request from the corresponding author.
